# The role of antimicrobial prophylaxis in laparoscopic nephrectomy for renal cell carcinoma

**DOI:** 10.1186/s12894-024-01447-2

**Published:** 2024-03-13

**Authors:** Mengchao Wei, Wenjie Yang, Weifeng Xu, Guanghua Liu, Yi Xie, Jie Dong, Zhigang Ji

**Affiliations:** Department of Urology, Peking Union Medical College Hospital, Chinese Academy of Medical Sciences and Peking Union Medical College, Dongcheng, Beijing, 100000 China

**Keywords:** Antibiotics, Laparoscopy, Nephrectomy, Renal cell carcinoma, Infection

## Abstract

**Background:**

To investigate the role of antimicrobial prophylaxis in laparoscopic nephrectomy for renal cell carcinoma.

**Methods:**

We retrospectively enrolled 1000 patients who underwent laparoscopic nephrectomy from August 2019 to November 2021 in the Peking Union Medical College Hospital. Patients were divided into group without antimicrobial prophylaxis (*n* = 444) and group with antimicrobial prophylaxis (*n* = 556). Outcomes including 30-day postoperative infection rate, the increase rate of pre- and post-operative white blood cell counts and hospital stay were analyzed.

**Results:**

The overall infection rate was 5.0% (28/556) in the group with antimicrobial prophylaxis, which was similar to 4.1% (18/444) in the group without antimicrobial prophylaxis (*P* = 0.461). The increase rate of pre- and post-operative white blood cell counts was significantly lower (85.5% versus 97.0%) in the group with antimicrobial prophylaxis (*P* = 0.004). The postoperative hospital stay was 5 (4, 6) days in both groups (*P* = 0.483). Logistic regression analyses identified the use of antimicrobial prophylaxis had no influence on the occurrence of infection events (odds ratio = 0.797; 95% confidence interval, 0.435–1.460; *P* = 0.462). Hemoglobin (odds ratio = 0.430; 95% confidence interval, 0.257–0.719; *P* = 0.001) and partial nephrectomy (odds ratio = 2.292; 95% confidence interval, 1.724–3.046; *P* < 0.001) influenced the use of antimicrobial prophylaxis independently.

**Conclusions:**

The use of antimicrobial prophylaxis had no impact on postoperative infection in patients receiving laparoscopic nephrectomy for renal cell carcinoma.

## Background

Antimicrobial prophylaxis (AMP) is widely used to prevent postoperative infection. In terms of urologic surgery, the European Association of Urology published a guideline for AMP in various surgical procedures [1]. However, little was demonstrated on laparoscopic urologic procedures. With the advantage of low invasiveness, laparoscopic surgery is popular in urologic field. Much evidence supports that the incidence of infection following laparoscopic surgery is lower than that of open surgery in either urologic field [2, 3] or non-urologic field [4–7]. Therefore, the protocols of perioperative AMP may be different between laparoscopic and open urologic surgery.

The Japanese Urological Association guideline recommends 1-day AMP protocol for clean operations and 3-day AMP protocol for clean-contaminated operations [8]. However, the guideline recommends the same AMP protocol for both laparoscopic and open urologic surgery. Yamamoto et al. pointed that more solid evidence is needed to establish a consensus for AMP use in laparoscopic procedures alone [9]. As early as 2004, Takeyama et al. demonstrated that the prophylactic efficacy of 1-day AMP was similar to that of 3-day AMP for clean or clean-contaminated urologic laparoscopic surgery [10]. Furtherly, Toshiki et al. prospectively investigated 373 cases undergoing gasless laparoendoscopic single-port surgery for renal or adrenal tumors without AMP use. It turned out that non-use of AMP and the on-demand use of antibiotics was efficient for minimally invasive renal and adrenal surgery [11]. In recent years, Aditya et al. presented that single‑dose cefuroxime was adequate for clean or clean-contaminated urologic surgery. However, only 65 patients underwent laparoscopic surgery in the study [12]. Therefore, it is possible that short-time AMP or even no AMP is sufficient to prevent postoperative infection for laparoscopic urologic surgery.

The incidence of renal cell carcinoma (RCC) is increasing, with approximately 400,000 new cases per year worldwide [13]. Laparoscopic nephrectomy is the standard treatment for localized RCC [14]. Radical nephrectomy is considered as clean surgery while partial nephrectomy is categorized as clean-contaminated surgery due to the possibility of urine contamination in the surgical field [9]. Until now, no standard AMP regimen for laparoscopic nephrectomy for RCC has been established. Inappropriate application of antibiotics during the perioperative period of RCC may result in the emergence of antimicrobial resistant bacteria, adverse events from administered drugs and the increase of hospital cost and stay [15, 16]. Therefore, there is an urgent need to demonstrate the role of AMP in laparoscopic nephrectomy for RCC.

Thus, we performed an investigation on the role of AMP in a Chinese tertiary center and tried to provide more evidence for deciding an optimal AMP regimen in laparoscopic nephrectomy for RCC.

## Methods

### Patients

We retrospectively enrolled 1000 patients who underwent laparoscopic nephrectomy from August 2019 to November 2021 in the Peking Union Medical College Hospital. The inclusion criteria were as follows: (1) patients were diagnosed as primary RCC by pathology; (2) patients underwent laparoscopic nephrectomy successfully, without conversion to open surgery; (3) patients were aged between 18 and 85 years. Patients were excluded if they had signs of infection prior to the operation, contaminated operation site or a history of autoimmune diseases requiring long-term immunosuppressant or steroid therapy. Patients were classified into AMP group and non-AMP group according to the use of AMP.

### Data collection

Patients’ clinical characteristics including sex, age, body mass index (BMI), history of smoking, hypertension, diabetes, cardiovascular disease, cerebral infarction and other malignancies, American Society of Anesthesiologists (ASA) score, hemoglobin (Hb), platelet (PLT), alanine aminotransferase (ALT), albumin (ALB), creatinine (CREA), side and size of the tumor lesion, surgical approach (radical or partial nephrectomy, retroperitoneal or transperitoneal), operation time and blood loss were collected. For patients in the AMP group, the duration of AMP and the type of the antibiotics were also collected.

### Operations and perioperative care

Robot-assisted or pure laparoscopic nephrectomy was performed via retroperitoneal or transperitoneal approach depending on the location and size of the tumor lesion. Usually three to five ports with skin incisions were needed. The maximum length of the incisions depended on the size of specimen. For radical nephrectomy, a skin incision approximately 8 cm in length was made to ensure enough space for hand insertion. All operations were performed under standard sterilization of the surgical sites. At the end of an operation, the surgical wound was closed by absorbable stitches, thereafter disinfected with iodine complex and then covered with sterile gauzes. Dressings were changed regularly after the operation.

### Outcomes

The primary outcome was 30-day postoperative infection rate, including surgical site infection (SSI) and remote infection (RI). SSI was categorized into superficial infection, deep infection and infection of organs/space, based on the guideline of SSI prevention [17]. RI was defined as systemic infection involving respiratory, urinary tract or gastrointestinal tract system. RI was featured by high fever (body temperature over 38.5℃), elevation of white blood cell (WBC) count and positive pathogenic tests, or no pathogenic evidence, but over two senior physicians reached an agreement of RI [18]. Secondary outcomes included the increase rate of pre- and post-operative WBC counts and hospital stay. For patients in the non-AMP group, the unplanned addition of antibiotics was also analyzed.

### Statistical analysis

Continuous variables including age, BMI, Hb, PLT, ALT, ALB, CREA, tumor size, operation time and blood loss were transformed into categorical variables based on normal reference values or clinical judgment. All categorical variables were presented as frequency and percentage. Difference test was conducted using the chi-square or Fisher’s exact test. The duration of AMP, increase rate of pre- and post-operative WBC counts and postoperative hospital stay were presented as median and interquartile range. The increase rate of WBC and postoperative hospital stay were compared using Mann-Whitney *U* test. Univariable and multivariable logistic regression analyses were used to determine factors influencing AMP using and postoperative infection. Statistical analyses were conducted using SPSS software (version 25, IBM). All tests were two-sided, and *P* < 0.05 indicated statistical significance.

## Results

### Patient characteristics

**Fig. 1:**
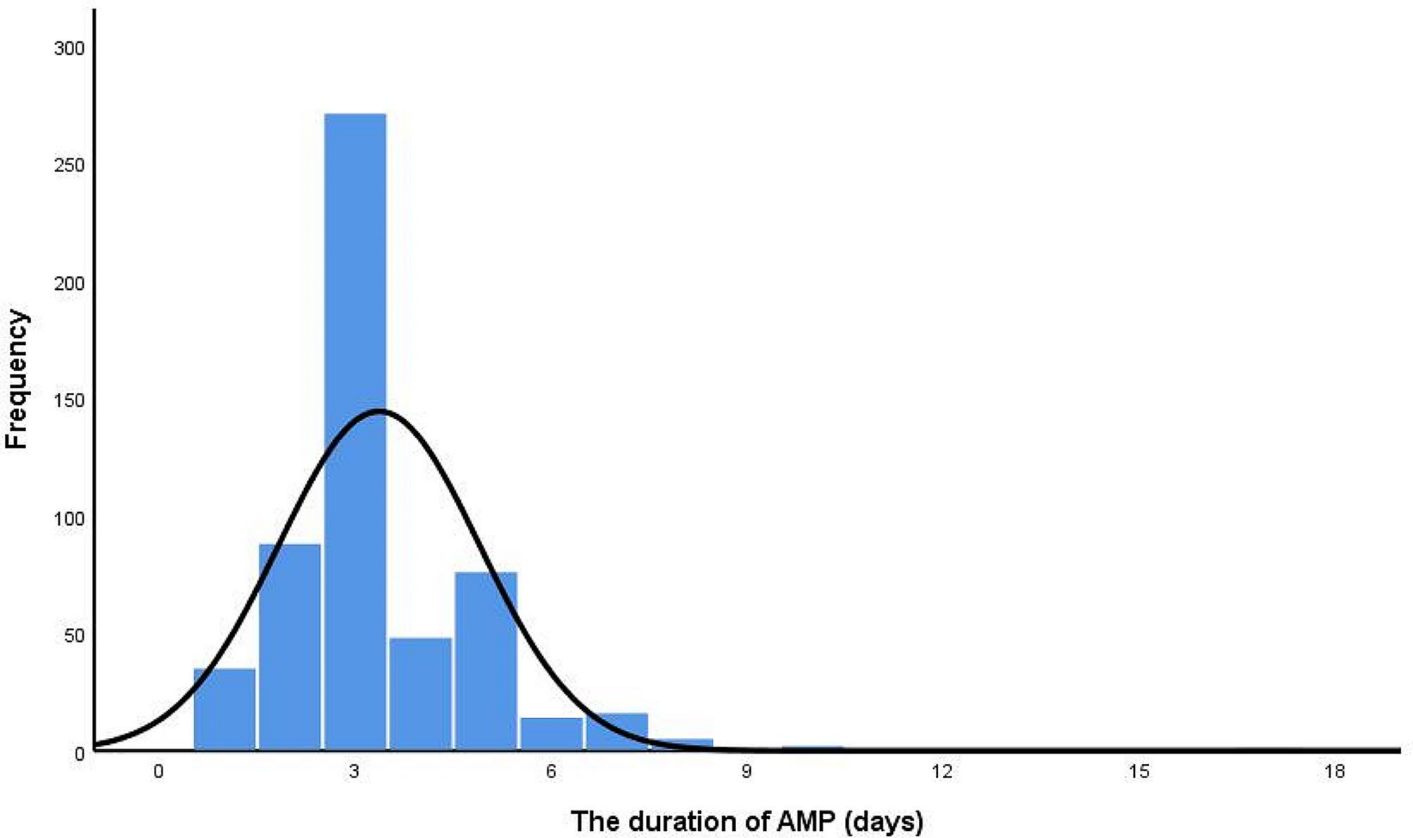
The distribution of AMP duration of all patients. Abbreviation: AMP, antimicrobial prophylaxis

A total of 1000 patients were included in the study, in which 556 patients received AMP and 444 patients did not. Baseline characteristics of the two groups were shown in Table 1. Patients in the AMP group were more likely to have anemia (for Hb less than reference value, 9.7% versus 5.4%, *P* = 0.012), smaller tumor lesions (for tumor size ≤ 7 cm, 94.8% versus 91.4%, *P* = 0.036), undergo partial nephrectomy (72.7% versus 54.3%, *P* < 0.001) and adopt transperitoneal approach (7.0% versus 4.1%, *P* = 0.045). Other characteristics were comparable between the two groups. For patients in the AMP group, the duration of AMP was 3 (3, 4) days. A proportion of 29.1% patients received AMP more than 3 days (Fig. 1). The types of antibiotics were shown in Table 2. Nearly 70% patients received second-generation cephalosporins.

**Table 1:** Baseline characteristics of the AMP group and non-AMP group

Characteristics	AMP (*n* = 556)	Non-AMP (*n* = 444)	*P* value
Sex (man/woman)	367(66.0)/189(34.0)	297(66.9)/147(33.1)	0.769
Age, years (> 60/≤60)	184(33.1)/372(66.9)	160(36.0)/284(64.0)	0.330
BMI, kg/m^2^ (> 24/≤24)	377(67.8)/179(32.2)	302(68.0)/142(32.0)	0.943
Smoking (yes/no)	183(32.9)/373(67.1)	141(31.8)/303(68.2)	0.698
Hypertension (yes/no)	239(43.0)/317(57.0)	181(40.8)/263(59.2)	0.480
Diabetes (yes/no)	112(20.1)/444(79.9)	74(16.7)/370(83.3)	0.160
Cardiovascular disease (yes/no)	66(11.9)/490(88.1)	48(10.8)/396(89.2)	0.600
Cerebral infarction (yes/no)	24(4.3)/532(95.7)	23(5.2)/421(94.8)	0.521
Other malignancies (yes/no)	55(9.9)/501(90.1)	40(9.0)/404(91.0)	0.636
ASA score (1/2)	230(41.4)/326(58.6)	200(45.0)/244(55.0)	0.243
Hb, g/L (more than reference value/less than reference value)^#^	502(90.3)/54(9.7)	420(94.6)/24(5.4)	0.012^*^
PLT, ×10^9^/L (> 100/≤100)	554(99.6)/2(0.4)	441(99.3)/3(0.7)	0.801
ALT, U/L (> 40/≤40)	55(9.9)/501(90.1)	34(7.7)/410(92.3)	0.218
ALB, g/L (≥ 28/<28)	555(99.8)/1(0.2)	444(100.0)/0(0.0)	1.000
CREA, µmol/L (more than reference value/less than reference value)^#^	18(3.2)/538(96.8)	14(3.2)/430(96.8)	0.940
Tumor side (left/right)	272(48.9)/284(51.1)	208(46.8)/236(53.2)	0.514
Tumor size, cm (> 7/≤7)	29(5.2)/527(94.8)	38(8.6)/406(91.4)	0.036^*^
Partial/Radical nephrectomy	404(72.7)/152(27.3)	241(54.3)/203(45.7)	< 0.001^*^
Surgical approach (retroperitoneal/transperitoneal)	517(93.0)/39(7.0)	426(95.9)/18(4.1)	0.045^*^
Operation time, min (> 120/≤120)	120(21.6)/436(78.4)	116(26.1)/328(73.9)	0.093
Blood loss, ml (> 100/≤100)	32(5.8)/524(94.2)	34(7.7)/410(92.3)	0.229

Abbreviations: AMP, antimicrobial prophylaxis; BMI, body mass index; ASA, American Society of Anesthesiologists; Hb, hemoglobin; PLT, platelet; ALT, alanine aminotransferase; ALB, albumin; CREA, creatinine

#The reference value of Hb is 120 g/L in man and 110 g/L in woman. The reference value of CREA is 104µmol/L in man and 84µmol/L in woman

*Statistically significant at α = 0.05

**Table 2:** The type of antibiotics used in the AMP group

Type	n (%)
Second-generation cephalosporins	395(71.0)
Third-generation cephalosporins	31(5.6)
Quinolones	94(16.9)
Clindamycin	3(0.5)
Piperacillin-tazobactam	1(0.2)
Second-generation cephalosporins and quinolones	23(4.1)
Second-generation cephalosporins and third-generation cephalosporins	4(0.7)
Second-generation cephalosporins and carbapenem	1(0.2)
Quinolones and piperacillin-tazobactam	1(0.2)
Second-generation cephalosporins and metronidazole	1(0.2)
Second-generation cephalosporins, carbapenem and quinolones	1(0.2)
Carbapenem, quinolones and metronidazole	1(0.2)

Abbreviation: AMP, antimicrobial prophylaxis

### Factors influencing the use of AMP

As shown in Table 3, univariable logistic regression analysis revealed that Hb, tumor size, partial nephrectomy and surgical approach were influencing factors for AMP. However, following multivariable logistic regression analysis indicated that only Hb [odds ratio (OR) = 0.430; 95% confidence interval (CI), 0.257–0.719; *P* = 0.001] and partial nephrectomy (OR = 2.292; 95% CI, 1.724–3.046; *P* < 0.001) influenced the use of AMP independently. Patients who had lower Hb and partial nephrectomy tended to receive AMP.

**Table 3:** Univariable and multivariable logistic regression analyses for factors influencing AMP

Characteristics	Univariable analysis			Multivariable analysis		
	β	OR (95% CI)	*P* value	β	OR (95% CI)	*P* value
Sex (man)	-0.040	0.961 (0.738, 1.252)	0.769			
Age, years (≤ 60)	0.130	1.139 (0.876, 1.480)	0.331			
BMI, kg/m^2^ (≤ 24)	0.010	1.010 (0.773, 1.319)	0.943			
Smoking (no)	-0.053	0.948 (0.726, 1.239)	0.698			
Hypertension (no)	-0.091	0.913 (0.709, 1.176)	0.480			
Diabetes (no)	-0.232	0.793 (0.573, 1.097)	0.161			
Cardiovascular disease (no)	-0.105	0.900 (0.606, 1.335)	0.600			
Cerebral infarction (no)	0.191	1.211 (0.674, 2.176)	0.522			
Other malignancies (no)	-0.103	0.902 (0.588, 1.383)	0.636			
ASA score (1)	0.150	1.162 (0.903, 1.495)	0.243			
Hb, g/L (more than reference value)^#^	-0.633	0.531 (0.323, 0.874)	0.013^*^	-0.844	0.430 (0.257, 0.719)	0.001^*^
PLT, ×10^9^/L (> 100)	0.634	1.884 (0.313, 11.327)	0.489			
ALT, U/L (≤ 40)	-0.281	0.755 (0.483, 1.181)	0.219			
ALB, g/L (≥ 28)	-20.980	0 (0, 0)	1.000			
CREA, µmol/L (less than reference value)^#^	-0.027	0.973 (0.479, 1.979)	0.940			
Tumor side (left)	0.083	1.087 (0.846, 1.395)	0.514			
Tumor size, cm (≤ 7)	0.531	1.701 (1.031, 2.805)	0.037^*^	0.101	1.106 (0.644, 1.899)	0.715
Partial nephrectomy	0.806	2.239 (1.720, 2.915)	< 0.001^*^	0.829	2.292 (1.724, 3.046)	< 0.001^*^
Surgical approach (retroperitoneal)	-0.580	0.560 (0.316, 0.993)	0.047^*^	-0.507	0.602 (0.336, 1.081)	0.089
Operation time, min (≤ 120)	0.251	1.285 (0.959, 1.722)	0.093			
Blood loss, ml (≤ 100)	0.306	1.358 (0.824, 2.238)	0.230			

Abbreviations: AMP, antimicrobial prophylaxis; OR, odds ratio; CI, confidence interval; BMI, body mass index; ASA, American Society of Anesthesiologists; Hb, hemoglobin; PLT, platelet; ALT, alanine aminotransferase; ALB, albumin; CREA, creatinine

#The reference value of Hb is 120 g/L in man and 110 g/L in woman. The reference value of CREA is 104µmol/L in man and 84µmol/L in woman

*Statistically significant at α = 0.05

### Outcomes

The overall 30-day postoperative infection rate was 5.0% (28/556) in the AMP group and 4.1% (18/444) in the non-AMP group. The difference of infection rates was not significant (*P* = 0.461). In the AMP group, 3 patients experienced SSI and 25 patients had RI. In the non-AMP group, 3 patients underwent SSI and 15 patients had RI. The detailed infection events were shown in Table 4. In terms of the secondary outcomes, the increase rate of pre- and post-operative WBC counts was 85.5% (58.0%, 121.8%) in the AMP group, significantly lower than 97.0% (67.3%, 124.8%) in the non-AMP group (*P* = 0.004). The postoperative hospital stay was 5 (4, 6) days in either AMP or non-AMP group (*P* = 0.483). For patients in the non-AMP group, the unplanned addition of antibiotics occurred in 5.0% (22/444) cases, in which 2 patients underwent SSI and 7 patients had RI. All the infection events were well-controlled by antibiotics. Of note, in the AMP group, the antibiotics was upgraded to control infection events in 1 patient with SSI and 4 patients with RI.

**Table 4:** Detailed infection events of the AMP group and non-AMP group

Infection events	Number of events	
	AMP group	Non-AMP group
SSI		
Superficial infection	1	1
Infection of organs/space	2	2
RI		
Respiratory infection	3	1
High fever without pathogenic evidence, but over two senior physicians reached an agreement of RI	22	14

Abbreviations: AMP, antimicrobial prophylaxis; SSI, surgical site infection; RI, remote infection

Additionally, we performed further subgroup analysis. In patients undergoing partial nephrectomy, the overall infection rate was 6.4% (26/404) in the AMP group and 5.0% (12/241) in the non-AMP group (*P* = 0.447). In patients undergoing radical nephrectomy, the overall infection rate was 1.3% (2/152) in the AMP group and 3.0% (6/203) in the non-AMP group (*P* = 0.504). In patients with operation time more than 120 min, the overall infection rate was 8.3% (10/120) in the AMP group and 5.2% (6/116) in the non-AMP group (*P* = 0.334). In patients with operation time within 120 min, the overall infection rate was 4.1% (18/436) in the AMP group and 3.7% (12/328) in the non-AMP group (*P* = 0.741). In patients with ASA 1, the overall infection rate was 3.5% (8/230) in the AMP group and 3.0% (6/200) in the non-AMP group (*P* = 0.780). In patients with ASA 2, the overall infection rate was 6.1% (20/326) in the AMP group and 4.9% (12/244) in the non-AMP group (*P* = 0.532). In the AMP group, the overall infection rate was 6.2% (10/162) in patients who received AMP more than 3 days and 4.6% (18/394) in patients who received AMP within 3 days (*P* = 0.432). Besides, the overall infection rate was 4.8% (19/395) in patients who received second-generation cephalosporins and 5.6% (9/161) in patients who received other types of antibiotics (*P* = 0.703).

### Factors influencing the occurrence of infection events

As shown in Table 5, univariable logistic regression analysis revealed that partial nephrectomy was the only influencing factor for the occurrence of infection events (OR = 2.715; 95% CI, 1.253–5.887; *P* = 0.011). Patients who underwent partial nephrectomy tended to experience infection events. The use of AMP had no influence on the occurrence of infection events (OR = 0.797; 95% CI, 0.435–1.460; *P* = 0.462).

**Table 5:** Univariable logistic regression analysis for factors influencing the occurrence of infection events

Characteristics	Univariable analysis		
	β	OR (95% CI)	*P* value
Sex (man)	0.152	1.165 (0.613, 2.213)	0.642
Age, years (≤ 60)	0.190	1.209 (0.636, 2.297)	0.563
BMI, kg/m^2^ (≤ 24)	-0.191	0.826 (0.429, 1.592)	0.569
Smoking (no)	0.320	1.377 (0.703, 2.696)	0.351
Hypertension (no)	-0.247	0.781 (0.432, 1.412)	0.413
Diabetes (no)	-0.576	0.562 (0.290, 1.091)	0.089
Cardiovascular disease (no)	0.351	1.421 (0.620, 3.256)	0.406
Cerebral infarction (no)	0.367	1.443 (0.431, 4.833)	0.552
Other malignancies (no)	0.569	1.766 (0.767, 4.067)	0.181
ASA (1)	0.570	1.767 (0.931, 3.355)	0.082
Hb, g/L (more than reference value)^#^	-0.644	0.525 (0.125, 2.208)	0.379
PLT, ×10^9^/L (> 100)	-18.176	0 (0, 0)	0.999
ALT, U/L (≤ 40)	0.234	1.263 (0.486, 3.282)	0.632
ALB, g/L (≥ 28)	-18.176	0 (0, 0)	1.000
CREA, µmol/L (less than reference value)^#^	-0.413	0.662 (0.088, 4.956)	0.688
Tumor side (left)	-0.175	0.839 (0.464, 1.518)	0.562
Tumor size, cm (≤ 7)	-1.207	0.299 (0.041, 2.203)	0.236
Partial nephrectomy	0.999	2.715 (1.253, 5.887)	0.011^*^
Surgical approach (retroperitoneal)	-0.297	0.743 (0.176, 3.145)	0.687
Operation time, min (≤ 120)	0.576	1.779 (0.952, 3.325)	0.071
Blood loss, ml (≤ 100)	-0.013	0.987 (0.298, 3.269)	0.983
Non-AMP	-0.227	0.797 (0.435, 1.460)	0.462

Abbreviations: OR, odds ratio; CI, confidence interval; BMI, body mass index; ASA, American Society of Anesthesiologists; Hb, hemoglobin; PLT, platelet; ALT, alanine aminotransferase; ALB, albumin; CREA, creatinine; AMP, antimicrobial prophylaxis

#The reference value of Hb is 120 g/L in man and 110 g/L in woman. The reference value of CREA is 104µmol/L in man and 84µmol/L in woman

*Statistically significant at α = 0.05

## Discussion

There has been inadequate evidence on the AMP use for laparoscopic nephrectomy up till now. Using a large-scale Chinese patient data, our study found that the nonuse of AMP had an equivalent effect on postoperative infection compared to AMP use in patients receiving laparoscopic nephrectomy for RCC.

We focused on a consecutive cohort undergoing laparoscopic nephrectomy and found that 44.4% patients received no AMP. The median duration of AMP was 3 days in AMP group, similar to the Japanese Urological Association guideline recommendation. The overall infection rate was 4.6% in the whole cohort, similar to the infection rates reported in previous studies [11, 19]. Our following analysis revealed that the infection rates of AMP and non-AMP groups were similar and AMP use had no effect on the occurrence of infection events, despite a less increase of pre- and post-operative WBC counts in the AMP group. Subgroup analysis showed that AMP use did not decrease infection rates in patients undergoing either partial nephrectomy or radical nephrectomy, despite partial nephrectomy was a risk factor for the occurrence of infection events. Besides, the addition and upgrade of antibiotics were adequate to control postoperative infection. Therefore, AMP may be not a necessary perioperative procedure in laparoscopic nephrectomy for RCC.

The possibility of eliminating AMP was also demonstrated in Toshiki’s study, though they only incorporated clean urologic surgeries [11]. The underlying reasons for the no impact of AMP on postoperative infection may lie in following aspects. First, except AMP use, measures including removal of hair, applying an incise drape to the surgical site, use of antimicrobial sutures and clean operating room environment also play an important role to prevent postoperative infection [15, 20]. Second, the smaller incision and less exposure of intracorporal oragans/tissues to the air in laparoscopic surgery may contribute to less infection events. Third, AMP was intended for prevention of SSI [21]. However, the incidence of RI was higher than that of SSI in our study. Therefore, the effect of AMP may be less obvious under above circumstances.

The nonuse of AMP for laparoscopic nephrectomy has several benefits. First, it may exert a preventive effect on the development of bacterial resistance. Misuse of antibiotics may increase bacterial resistance [22, 23]. Calvert et al. reported that extended AMP beyond 24 h for partial or radical nephrectomy was associated with a 3.79 times higher possibility of Clostridium difficile infection [24]. In our study, nearly 30% patients in the AMP group received AMP for more than 3 days and AMP regimens varied in different types of antibiotics, which to some extent mirrors the abuse of antibiotics. Therefore, there is an urgent need to cut down unnecessary AMP use in laparoscopic nephrectomy. Second, the reduced use of AMP may help lower the risk of adverse events associated with antibiotics, such as allergic reaction, gastrointestinal reaction and damage to hepatic/renal function. Although the adverse events following antibiotics administration are relatively uncommon, it remains valuable to prevent the occurrence of adverse events since it’s difficult to evaluate and predict the degree of each adverse event [25]. Third, the reduced use of AMP can relieve the socioeconomic burden caused by the expense of antibiotics, instruments used for administration, as well as labor cost [22, 26–28]. This benefit is even prominent in China owing to its large population and relatively limited medical resources.

Our analysis also revealed that patients with patients who had lower Hb and partial nephrectomy tended to receive AMP. It’s reasonable for physicians to apply AMP in patients undergoing partial nephrectomy since partial nephrectomy is seen as clean-contaminated. Anemia usually reflects a status of malnutrition, which is a risk factor for perioperative infections [8]. Therefore, physicians tended to use AMP in patients with lower Hb.

To our knowledge, this is the largest cohort study focusing on the AMP use in laparoscopic nephrectomy for RCC. Our study indicated that in patients undergoing laparoscopic nephrectomy for RCC, eliminating AMP use may be possible. However, there are several limitations in our study. First, the study is retrospective and non-randomized. There remains a risk of selection and confounding bias. Randomized controlled trials are needed to provide more solid evidence on this issue. Second, our study is only suitable for patients with relatively good general conditions. For patients with high-risk factors for infection, such as severe diabetes, immunocompromised status and other severe comorbidities, the use of AMP should be cautiously assessed.

## Conclusions

In conclusion, the nonuse of AMP had an equivalent effect on postoperative infection compared to AMP use in patients receiving laparoscopic nephrectomy for RCC. AMP may be not a necessary perioperative procedure in laparoscopic nephrectomy for RCC.

## Data Availability

The datasets used and/or analysed during the current study are available from the corresponding author on reasonable request.

## Abbreviations

AMP: Antimicrobial prophylaxis
ALB: Albumin
ALT: Alanine aminotransferase
ASA: American Society of Anesthesiologists
BMI: Body mass index
CI: Confidence interval
CREA: Creatinine
Hb: Hemoglobin
OR: Odds ratio
PLT: Platelet
RCC: Renal cell carcinoma
RI: Remote infection
SSI: Surgical site infection
WBC: White blood cell
